# The Ufm1 Cascade

**DOI:** 10.3390/cells3020627

**Published:** 2014-06-11

**Authors:** Jens Daniel, Eva Liebau

**Affiliations:** Department of Molecular Physiology, Westfälische Wilhelms-University Münster, Schlossplatz 8, D-48143 Münster, Germany; E-Mail: j_dani02@uni-muenster.de

**Keywords:** Ufm1, UBL, ER stress, unfolded protein response

## Abstract

The ubiquitin-fold modifier 1 (Ufm1) is a posttranslational modifier that belongs to the ubiquitin-like protein (UBL) family. Ufm1 is present in nearly all eukaryotic organisms, with the exception of fungi. It resembles ubiquitin in its ability to be ligated to other proteins, as well as in the mechanism of ligation. While the Ufm1 cascade has been implicated in endoplasmic reticulum functions and cell cycle control, its biological role still remains poorly understood. In this short review, we summarize the current state of Ufm1 research and its potential role in human diseases, like diabetes, ischemic heart disease and cancer.

## 1. Introduction

Posttranslational modifications are cellular mechanisms that enable a rapid response to internal and external changes. Beside small molecule modifications, like acetylation, methylation or phosphorylation, a large family of small proteins (e.g., ubiquitin and ubiquitin-like proteins) has evolved that is covalently attached to and detached from other proteins, thereby modifying their function [1]. In this review, we will focus on Ufm1 (ubiquitin-fold modifier 1), an ubiquitin-like protein (UBL) that was discovered 12 years ago [2,3], and summarize the current state of knowledge. Since the use of different terms for the same proteins should be avoided, we propose a unified nomenclature (Table 1).

**Table 1:** Proposed names of the Ufm1 system and their aliases.

Proposed Names	Aliases
Ufm1 (ubiquitin-fold modifier 1)	C13orf20
Uba5 (ubiquitin-like modifier activating enzyme 5)	UBE1DC1
Ufc1 (ubiquitin-fold modifier conjugating enzyme 1)	HSPC155
Ufl1 (Ufm1-specific ligase 1)	KIAA0776, NLBP, Maxer, RCAD
UfSP1 (Ufm1-specific protease 1)	Inactive Ufm1-specific protease 1
UfSP2 (Ufm1-specific protease 2)	C4orf20
Ufbp1 (Ufm1-binding protein 1 containing a PCI domain)	C20orf116, Dashurin, DDRGK domain containing 1
Cdk5rap3 (CDK5 regulatory subunit-associated protein 3)	LZAP, protein HSF-27, IC53, C53, ARF-binding protein

## 2. The Ufm1 Cascade

Ufm1 is a 9.1-kDa protein with a similar tertiary structure to ubiquitin [2]. Like other UBLs, it has a low sequence identity to ubiquitin, but shares its β-grasp fold. In contrast to ubiquitin and other UBLs, Ufm1 has a nonpolar surface, indicating a different set of interacting partners [3].

Ufm1 is present in nearly all eukaryotic organisms with the exception of fungi. This has led to the hypothesis that the cascade has evolved two times separately [3]. Another explanation could be the loss of the cascade in fungi. The nucleotide sequence of Ufm1 is highly conserved, with about 6% differences between vertebrates and less than 20% differences between vertebrates and plants.

**Figure 1:**
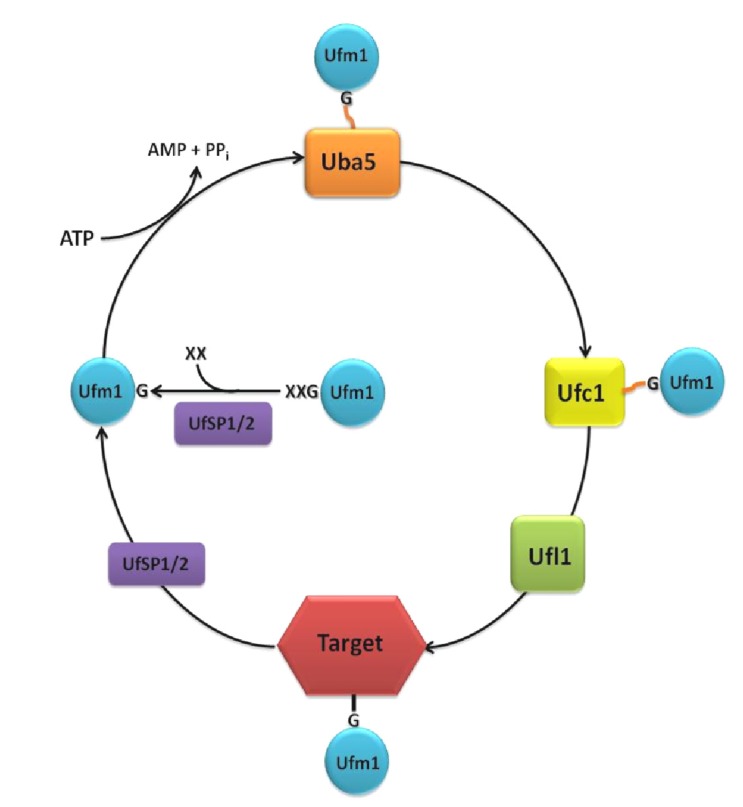
The Ufm1 cascade. The removal of one or two amino acids (XX) at the C-terminus is a prerequisite for the conjugation of Ufm1 to its targets. The maturation of the pro-form of Ufm1 is processed by the proteases, UfSP1 or UfSP2, exposing a conserved glycine (G). Ufm1 is activated in an ATP-dependent reaction by the E1 activating enzyme, Uba5, creating a thioester-bond between the C-terminal glycine of Ufm1 and the active cysteine residue of Uba5. Ufm1 is then transferred to the active cysteine of the E2 conjugating enzyme, Ufc1. With the aid of the E3 ligase, Ufl1, an isopeptide bond between Ufm1 and the target protein is established. Deconjugation is also catalyzed by UfSP1 or UfSP2.

Unlike ubiquitin and other UBLs, like the small ubiquitin-related modifier (SUMO), Ufm1 possesses a single active glycine at the C-terminus, which is required for the covalent attachment to its target proteins [3]. Depending on the species, one or two additional amino acids are appended to the C-terminal glycine. The precursor protein is cleaved by the two Ufm1-specific proteases, UfSP1 and UfSP2, to generate mature Ufm1 [4] (Figure 1).

Ufm1 is activated by the E1 ubiquitin-like modifier activating enzyme, Uba5. In the first step, the adenylated Ufm1 forms a noncovalent complex with Uba5. In the second step, Ufm1 is attached to the Uba5 catalytic cysteine via a thioester bond [5]. Uba5 is a member of the ubiquitin-activating protein family (UBA) and the only known E1 enzyme of the Ufm1 cascade. This family is characterized by two catalytic half-domains, the FCCH (first catalytic cysteine domain) and the SCCH (second catalytic cysteine domain), which mediate the binding of the E2 enzyme and the transfer of the activated UBL in a similar thioester linkage. However, Uba5, which is significantly smaller than any other UBA family member, does not possess these characteristic domains. Instead, Uba5 relies on a unique position of the catalytic cysteine in an α-helix motif of the adenylation domain and on conformational changes associated with the binding of ATP [5]. While *in vitro* assays confirm the activity of monomeric Uba5 [3], ultracentrifugation analysis indicates that Uba5 is likely active as a dimer [5]. Therefore, the possible dimerization remains elusive.

Interestingly, Zheng *et al.* [6] demonstrated the activation of the UBL, Sumo2, by Uba5. While activation of Ufm1 takes place in the cytosol, Sumo2 is possibly activated and/or transferred to the nucleus by Uba5 [6]. The unusual case of an E1 enzyme being able to activate more than one UBL has been described for the autophagy-related APG7 [7]. However, Tatsumi *et al.* [8] demonstrated that a loss of Uba5 had no effect on any UBL conjugation, except for Ufm1.

Following Ufm1 activation, the ubiquitin-fold domain of Uba5 interacts with the α2-helix of the Ufm1 conjugating enzyme 1 (Ufc1) [9] (Figure 1). In a transesterification reaction, the activated Ufm1 is then transferred from Uba5 to the Cys116 of Ufc1 [3,5]. Although Ufc1 does not share much sequence identity with other E2 enzymes, a catalytic E2 core domain consisting of 10 amino acids was identified. Therefore, it is concluded that the observed conjugation mechanism is similar to that of other E2 reactions [9,10,11].

The ligation of Ufm1 to target proteins is mediated by the E3 Ufm1-ligating enzyme 1 (Ufl1). Ufl1 recruits Ufc1, as well as the target proteins, like the Ufm1-binding protein 1 (Ufbp1), with its N-terminal region (Figure 1). Ufl1 stimulates the transfer of Ufm1 to the target protein via the generation of an ε-amid bonding between the C-terminal glycine of Ufm1 and the Lys267 of Ufbp1 [8]. Since Ufl1 does not possess a HECT-type catalytic domain or a RING finger domain, it cannot be classified into any of the known E3 enzyme classes [8,12].

Ufbp1 is a highly conserved protein with unknown function, found only in multicellular organisms. Ufbp1 was identified as a target of Ufm1, with ufmylation taking place in the PCI domain (Lys267). The PCI domain is a known protein-protein interaction mediator involved in several multiprotein complexes, like the 26S proteasomal “lid”, the COP9 signalosome (CSN) and the eukaryotic translation initiation factor 3 (eIF3), which regulate the protein life span [13,14]. Recently, Neziri [13] demonstrated that Ufbp1 is not associated with the proteasome complex. Instead, the hydrophobic N-terminal region anchors Ufbp1 to the cytosolic side of the endoplasmic reticulum (ER) membrane. Here, it recruits UfSP2 and colocalizes with Ufl1 and the Ufm1-target CDK5rap3 in a large multi-protein complex [15]. The presence of Ufbp1 enriches Ufm1 in the ER. An overexpression of Ufbp1 was reported to mediated ER proliferation and neogenesis [16].

The highly conserved Cdk5rap3 (Cdk5 activator-binding protein C53) is engaged in various cell signaling pathways that are involved in tumorigenesis and metastasis [17]. Cdk5rap3 has been proposed to be a tumor suppressor due to the inhibition of the nuclear factor κB (NF-κB) pathway [18]. The NF-κB pathway is a major player in the regulation of diverse biological processes, including development, immune responses, cell proliferation and apoptosis. Defects in NF-κB have been linked to a variety of human diseases, particularly cancers [19]. NF-κB resides in the cytoplasm in an inactive form that is associated with inhibitory proteins, termed I-κB, the most important ones being I-κBα, I-κBβ and I-κBε. Upon phosphorylation and ubiquitin-dependent degradation of I-κBα, NF–κB translocates to the nucleus and functions as a transcription factor [20].

As mentioned above, the processing, as well as the deconjugation of Ufm1 is mediated by the two Ufm1-specific proteases, UfSP1 and UfSP2. Kang *et al.* (2007) and Ha *et al.* (2008) show that these cysteine proteases, which have a catalytic triad consisting of cysteine, histidine und aspartic acid, do not exhibit any obvious homologies to other proteases and deubiquitinating enzymes (DUBs) [4]. Although the conserved sequences around the catalytic motifs are broadly present in UfSP1 family members, UfSP1 is wrongly classified as non-functional in many organisms. The smaller UfSP1, exhibit a higher enzymatic activity in *in vitro* assays compared to the larger UfSP2 [4]. However, since it is only present in placenta animals, where it is usually expressed weaker than UfSP2, a subordinated role is assumed. The prolonged N-terminal region of UfSP2 is proposed to be a mediator for substrate specificity in the deconjugation process. Interaction of this N-terminal region with the Ufm1 target Ufbp1 was proposed to recruit Ufsp2 to the ER membrane [15].

Interestingly, UfSP2 is reported to have a distinct neuronal expression in *Caenorhabditis elegans*, whilst other Ufm1 cascade members are mainly expressed in the intestine. This might point to a non-essential role in the maturation of Ufm1. A recent study demonstrated an Ufm1-independent function of UfSP2 in the maturation of G-protein coupled receptors at the ER membrane [21].

## 3. The Role of Ufm1 in ER Homeostasis

The Ufm1 cascade has been studied in mammals, *C. elegans* and the protozoan parasite, *Leishmania donovani*. With the exception of *L. donovani*, its activity has been linked to the ER [16,22,23,24,25,26].

The ligase, Ufl1, UfSP2 and the target proteins, Ufbp1 and Cdk5rap3, were shown to aggregate in a large protein complex at the cytosolic side of the ER membrane [16,23,25,27]. Components of the Ufm1 cascade have been demonstrated to be induced specifically under ER stress [22]. Moreover, in mice, Ufm1 was shown to be a direct target of the transcription factor, XBP1 (X-box binding protein 1) [28]. XBP1 is a major activator of the unfolded protein response (UPR) [29], and components of the Ufm1 cascade contain consensus XBP1-binding elements (UPRE) in their promoter region [30]. Accordingly, the Ufm1 cascade is especially expressed in cells with increased ER stress, like ischemic heart cells or hibernating squirrel brain cells, as well as in protein-secreting cells, like the pancreatic beta cells [22,31].

Loss of function of the Ufm1 cascade in mice leads to apoptosis in fetal liver cells and pancreatic beta cells [23,32]. The death of pancreatic beta cells was shown to be mediated by a specific ER stress-induced apoptosis program [23]. Consequently, Tatsumi *et al.* [28] came to the conclusion that the Ufm1 cascade is involved in vesicle trafficking with the loss of function leading to an increased protein load and resulting in enhanced ER stress. However, in *C. elegans*, where ER stress-induced apoptosis does not occur in adult animals, the UPR is induced and mediates a higher stress resistance in the absences of the Ufm1 cascade [24]. Interestingly, Lemaire *et al.* [23] showed that Ufm1 conjugation is high when the protein load is low and *vice versa*. These results suggest an involvement of the Ufm1 cascade in the homeostasis of the ER stress response. In *C. elegans*, the regulatory effects of the Ufm1 cascade were also observed for other stressors, like oxidative, heat and pathogen stress. The loss of function of the Ufm1 cascade leads to increased survival in the presence of these stressors. Interestingly, it also leads to an increased susceptibility for heavy metal stress [24].

## 4. Ufm1 and Cell Differentiation

The Ufm1 cascade was shown to be essential for the differentiation of erythroid progenitors. In the embryonic liver of Uba5^−/−^ knock-out mice, the differentiation of both megakaryocytes and erythroids is impaired and apoptosis is increased. The resulting anemia is considered to be the main cause of death in Uba5^−/−^-deficient mice embryos. Although a cell-specific rescue of Uba5 stopped anemia, prenatal death could not be prevented, indicating the additional essential roles of the Ufm1 system [8].

Recently, a mutation within the human UfSP2 has been identified to be associated with Beukes familial hip dysplasia, an autosomal dominant disorder characterized by premature degenerative osteoarthritis of the hip joint [15]. The expression of the Ufm1 cascade was shown to be upregulated during osteogenic and chondrogenic differentiation. This coincided with the induction of the UPR [30].

**Figure 2:**
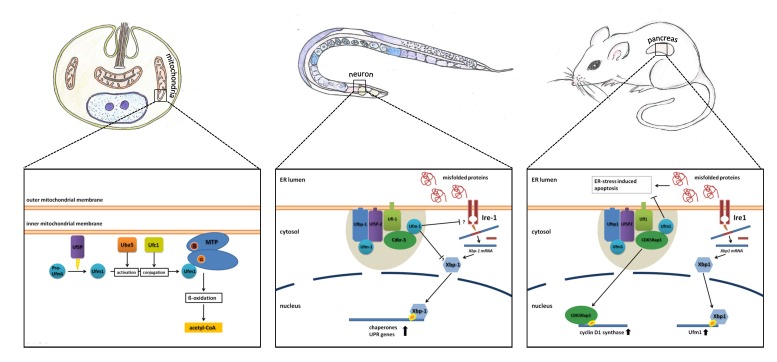
Simplified model of the known functions of the Ufm1 cascade in *L. donovani*, *C. elegans* and mammals. The known implications of the Ufm1 cascade are discussed in the text.

Hertel *et al.* [24] proposed that in the absence of the Ufm1 cascade, the observed defects in cell differentiation are caused by ER stress-induced apoptosis (Figure 2).

## 5. Ufm1 and Cell Cycle Control

The Ufm1 cascade is also involved in cellular growth and development. The ligating enzyme, Ufl1, was shown to interact with CDK5Rap3, a tumor suppressor that regulates the cyclin D1 synthase [25,27]. Ufl1 is able to change the localization and stability of CDK5Rap3 by recruiting it to a large multi-protein complex at the cytosolic site of the ER plasma membrane and protecting it from proteasomal degradation [16]. Through this interaction, Ufl1 was reported to regulate the NF-κB pathway and cell invasion [16,25]. Accordingly, Ufl1 was reported to be decreased in several tumor tissues and increased in cells lines, with little cell invasion [25]. However, the role as a tumor suppressor is controversial due to its increased expression in human lung adenocarcinoma. Furthermore, overexpression of Ufl1 promotes proliferation by inhibiting the proteasome-mediated degradation of p120 catenin [33]. Taken together, Ufl1 plays a protective role against proteasome-mediated degradation of proteins. Although it is likely that Ufl1 protects these proteins by ufmylation, this still needs to be verified.

In addition, Ufbp1 regulates the stability of I-κBα in the NF-κB pathway. A deletion of Ufbp1 leads to a decreased cell proliferation and invasion in human cells [34]. Ufbp1 seems to affects the stability of I-κBα, by interfering with its ubiquitylation and phosphorylation site [34].

In summary, different components of the Ufm1 system aggregate in a large protein complex at the cytosolic site of the ER membrane. Here, they are involved in the regulation of the cell cycle by influencing the stability of NF-κB binding proteins [16,25,34] (Figure 2).

## 6. Ufm1 in Different Model Organisms

The Ufm1 cascade was investigated in the model organism, *C. elegans*, in terms of function, and the interactions of the involved proteins were characterized *in vitro* and *in vivo*. The loss of function of the Ufm1 cascade leads to a decreased rate of larval development and reproduction. Since mutants also displayed a widely increased resistance against various stressors, like ER, oxidative, heat and pathogen stress, a shift in energy metabolism was postulated [24].

Knockout of the Ufm1 cascade leads to an induction of the UPR in both mammals and nematodes. At the same time, this loss of function promotes ER stress-induced apoptosis in mammals. In *C. elegans*, where apoptosis does not occur in adult animals, the UPR seems to work more efficient in the absence of the Ufm1 cascade. Additionally, the BIP homologue, Hsp-4, was induced, pointing to an increased IRE1-dependent UPR. Therefore, Hertel *et al.* [24] came to the conclusion that the elevated UPR and not increased ER stress is responsible for the observed apoptosis events in mammals (Figure 2).

The Ufm1 cascade has also been investigated in the blood-borne pathogen, *L. donovani* [26,35,36,37]. Interestingly, the cascade is localized in the mitochondria, and the only known target protein is not a homologue of Ufbp1, but the mitochondrial trifunctional protein (MTP) [26]. This protein is attached to the inner mitochondrial membrane and catalyzes three consecutive steps in the beta oxidation of long-chain acyl-CoA esters. Through ufmylation of MTP, the amount of CoA-production in the beta oxidation is regulated. Decreased ufmylation therefore leads to a decrease in beta oxidation and affects energy metabolism, resulting in impaired growth and the reduced survival of amastigotes, the parasitic stage that is present in humans [26]. UfSP, the singular Ufm1-specific protease found in *L. donovani*, was shown to be essential for Ufm1 maturation. Deletion of UfSP displayed similar effects; furthermore, mouse infection experiments resulted in reduced virulence of the UfSP^−/−^ parasites [41]. This led Gannavaram *et al.* [41] to postulate that the Ufm1-associated enzymes could be exploited as anti-leishmanial drug targets (Figure 2).

## 7. Diseases Associated with the Ufm1 Cascade

The involvement of the Ufm1 cascade in diseases is diverse. Reports include ischemic heart diseases [22], diabetes [23], gastric lesions [38], schizophrenia [39], hip dysplasia [15] and cancer [25,33].

A strong upregulation of Ufm1 was observed in a mouse model of ischemic heart disease that is induced by chronic inflammation. In this model system, cardiomyocytes are exposed to proinflammatory cytokines, altered redox state and oxidative stress, which trigger the ER stress response. Activation of the UPR is also associated with the development of ischemic heart disease in humans. The elevated expression of Ufm1 was associated with the increased ER stress of the cardiomyocytes [22].

Lemaire *et al.* [23] showed that Ufm1 and its target, Ufbp1, are highly expressed in the insulin-secreting beta cells localized in the pancreatic islets of Langerhans in mice. Due to their secretory functions, beta cells have a highly developed ER and a high protein load, making them vulnerable to ER stress. Here, it was shown that Ufm1 and Ufbp1 protect the cells from ER stress-induced apoptosis [23]. The link between ER dysfunction and diabetes have been investigated in great detail [40].

A recent study discovered the association of Ufm1 single-nucleotide polymorphisms (SNPs) with gastric cancer and *Helicobacter pylori* infection in a low risk population in Malaysia. During the stage of atrophic gastritis, Ufm1 expression reflects the secretory status of the gastric mucosa [38].

Rubio *et al.* [39] report on significant disruptions of the ubiquitin/UBL conjugation in the pathophysiology of schizophrenia. A recent study linked ER stress to schizophrenia [41]. Rubio *et al.* [39] associated the decreased expression of components of the Ufm1 cascade observed in schizophrenic patients with ER stress alterations.

A mutation in the UfSP2 gene could be determined as a cause of Beukes familial Hip Dysplasia (BHK). This disease leads to premature degenerative osteoarthropathy of the hip joint. A role of the Ufm1 cascade in ossification processes is discussed [30].

Ufl1 was shown to be involved in the neurodegenerative disorder, spinocerebellar ataxia type 1 (SCA1), which belongs to the polyglutamine diseases. Shiwaku *et al.* [27] suggest that Ufl1 deficiency contributes to SCA1 pathology through the functional deficiency of Bergmann glia. Here, it has a critical role in G1/S transition and regulates cell proliferation through the regulation of the nuclear translocation of CDK5RAP3 [27].

It has been shown that components of the Ufm1 cascade are upregulated under ER stresses in multiple cancer cell lines. In a study by Kwon *et al.* [25], an important role of Ufl1 and CDK5RAP3 in tumorigenesis was reported. The interaction of these proteins was demonstrated to be necessary for tumor suppression through the inhibition of cell invasion and NF-κB activation. However, since Ufl1 is highly expressed in the early stage of lung adenocarcinoma and was reported to be associated with the proliferation of these tissues, its role in tumorigenesis is still controversial [33].

## 8. Conclusions

Research on the Ufm1 cascade is still in its infancy, and its functions are not yet completely understood. However, the Ufm1-associated proteins are highly conserved throughout eukaryotes, indicating their importance in cellular homeostasis. The significance of the Ufm1 cascade is also underlined by its implication in various human diseases. In general, the cascade seems to be involved in cellular homeostasis, influencing cell division, growth and ER function.

## Abbreviations

APG: Autophagy proteins
BiP: Binding immunoglobulin protein
CDK5rap3: CDK5 regulatory subunit-associated protein 3
CSN: COP9 signalosome
DUBs: Deubiquitinating enzymes
EIF3: Eukaryotic translation initiation factor 3
ER: Endoplasmic reticulum
FCCH: First catalytic cysteine half-domain
HECT: Homologous to the E6-AP carboxy terminus
I-KB: Inhibitor of κB
IRE1: Inositol-requiring protein 1
MTP: Mitochondrial trifunctional protein
NF-κB: Nuclear factor-κB
PCI: Proteasome, COP9, Initiation factor-3
RING: Really interesting new gene
SCCH: Second catalytic cysteine half-domain
SNPs: Single nucleotide polymorphisms
SUMO: Small ubiquitin-like modifier
UBA: Ubiquitin-associated domain
Uba5: Ubiquitin-like modifier activating enzyme 5
UBL: Ubiquitin-like proteins
Ufbp: UFM1-binding protein 1 containing a PCI domain
Ufc1: Ubiquitin-fold modifier conjugating enzyme 1
Ufl1: Ufm1-specific ligase 1
Ufm1: Ubiquitin-fold modifier 1
UfSP: Ufm-specific peptidase
UPR: Unfolded protein response
XBP1: X-box binding protein 1
